# Genetic Diversity of the Edible Mushroom *Pleurotus* sp. by Amplified Fragment Length Polymorphism

**DOI:** 10.1007/s00284-012-0175-7

**Published:** 2012-07-06

**Authors:** Anna Pawlik, Grzegorz Janusz, Joanna Koszerny, Wanda Małek, Jerzy Rogalski

**Affiliations:** 1 Department of Biochemistry, Maria Curie-Sklodowska University, Akademicka 19, 20-033 Lublin, Poland; 2 Department of Genetics and Microbiology, Maria Curie-Sklodowska University, Akademicka 19, 20-033 Lublin, Poland

## Abstract

*Pleurotus* strains are the most important fungi used in the agricultural industry. The exact characterization and identification of *Pleurotus* species is fundamental for correct identification of the individuals and exploiting their full potential in food industry. The amplified fragment length polymorphism (AFLP) method was applied for genomic fingerprinting of 21 *Pleurotus* isolates of Asian and European origin. Using one *Pst*I restriction endonuclease and four selective primers in an AFLP assay, 371 DNA fragments were generated, including 308 polymorphic bands. The AFLP profiles were found to be highly specific for each strain and they unambiguously distinguished 21 *Pleurotus* sp. fungi. The coefficient of Jaccard’s genome profile similarity between the analyzed strains ranged from 0.0 (*Pleurotus* sp. I vs. *P. sajor*-*caju* 237 and *P. eryngii* 238) to 0.750 (*P. ostreatus* 246 vs. *P. ostreatus* 248), and the average was 0.378. The AFLP-based dendrogram generated by the UPGMA method grouped all the *Pleurotus* fungi studied into two major clusters and one independent lineage located on the outskirt of the tree occupied by naturally growing *Pleurotus* species strain I. The results of the present study suggest the possible applicability of the AFLP-*Pst*I method in effective identification and molecular characterization of *Pleurotus* sp. strains.

## Introduction

The genus *Pleurotus* (Jacq.: Fr.) Kumm. (*Pleurotaceae*, higher *Basidiomycetes*) comprises a cosmopolitan group of mushrooms with high nutritional value, therapeutic properties, and various environmental and biotechnological applications [7]. These morphologically distinct fungi consist of microorganisms that share the common character, i.e., the ability to produce arthrospores from asexual fructifications on basidiomata and/or in mycelial cultures [38]. Several species of the genus *Pleurotus* have great commercial value in the global market of edible cultivated mushrooms, which reportedly grew from 2.13 million tons in 1996 to 3.43 million tons in 2007 [26]. In line with this, the production of oyster mushrooms is also increasing; they currently rank third behind *Agaricus bisporus* (J. Lge) Imbach and *Lentinula edodes* (Berk.) Pegler in annual production of 875,600 tons [6]. Beside unquestionable importance in the worldwide agriculture and food industry, all species belonging to the genus *Pleurotus* are producers of several enzymes including ligninolytic ones such as laccase and peroxidases [22, 27]. Traditionally, edible species of the genus *Pleurotus* were considered as medicinally important mushrooms [1, 9]. Nowadays, nearly every paper concerning these fungi brings to light the new bioactive substances exhibiting antibiotic, antiviral, antitumor, and anticholesterolic activities. These pharmaceutical substances have been purified and characterized, making them biotechnologically important agents that should be investigated clinically [7, 21, 28]. The genus *Pleurotus* is also one of the most diverse groups among cultivated fungi with many taxonomic problems [35]. According to Zervakis and Balis [37], the taxonomic disagreements in the genus *Pleurotus* have risen for the following reasons: initial misidentification, absence of type specimens, instability of morphological characters due to environmental changes, limited reports on physiological characteristics, and lack of mating compatibility studies. Thus, to clarify the taxonomic status of species in the genus *Pleurotus* (earlier determined mainly by morphological features), many researchers started to classify these fungi also by genomic criteria [2].

As all other cultivated fungi, the cultivated lines of *Pleurotus* sp. can undergo a drastic loss of diversity resulting from man’s selection [11, 14]. This genetic erosion increases genetic vulnerability and it may lead to dramatic effects on the production yield during cultivation [8]. The development of tools aimed at the clear-cut and safe identification, and assessment of genetic variability of the wild and cultivated strains is thus a fundamental goal of molecular genetics research [33]. The crucial challenge in developing new methods for tracking microorganisms and their identification is to acquire rapidly markers with a high level of discriminative power and interlaboratory reproducibility for specific and sensitive detection of a target organism in complex environmental or commercial samples. Markers based on amplified fragment length polymorphisms (AFLP) have potential in this respect [13, 36].

AFLP is a PCR-based technique for genotyping and fingerprinting DNA of any origin and complexity, i.e., human, plant, and microbial [3, 23, 29, 31, 34]. A simplified AFLP protocol was developed for rapid genomic characterization of genomic DNA. The major modifications to the standard AFLP procedure included: one-step digestion–ligation reaction with digestion with a single restriction endonuclease and amplification in one reaction [29, 32]. Reproducibility, reliability, and specificity are the main advantages of the AFLP technique, which has already been applied to establish differences among *Pleurotus eryngii* strains [17, 18, 24, 33].

In this paper, we describe the usefulness of a simplified AFLP technique for studying genomic diversity and for identification of *Pleurotus* sp. strains.

## Materials and Methods

### Fungal Strains and Their Cultivation

*Pleurotus* strains (Table 1) were obtained from different collections: the Laboratory for Anatomy and Physiology of Plants, the I.E. Purkyne University, Brno, Slovakia (LAPU); Agriculture University, Tokyo, Japan (FAT, FCTUA); The Fungal Collection of Lublin, the Department of Biochemistry, Maria Curie-Sklodowska University, Lublin, Poland (FCL); and the Botany Institute II, Regensburg University, Regensburg, Germany (BIUR). Environmental fungal isolate (*Pleurotus* sp. I) was collected in Lublin, Poland. Commercially available *Pleurotus ostreatus* II and III were derived from local mushrooms producers (L. W. Skrzypczyk and H. Kaczmarek mushroom farms, respectively), Poland. The stock culture of fungal strains was maintained on GPY slants (glucose 1 g/l, peptone 0.5 g/l, yeast extract 0.1 g/l, agar 20 g/l). The slants were inoculated with mycelia and incubated at 26 °C for 7 days, and then used for seed culture inoculation. The mycelia of *Pleurotus* strains were transferred into a 100 ml Erlenmeyer flask containing 40 ml stationary liquid Lindeberg–Holm (LH) medium [19] by punching out about 5 mm^2^ of the slants with a sterilized cutter. The seeds were cultivated for 14 days at 26 °C. Broth cultures were then harvested by centrifugation at 10,000×*g* for 10 min and used for DNA extraction.

**Table 1:** List of fungal strains used in this study

Strain number in FCL^a^	Strain name	Strain source/other collection^b^
13	*Pleurotus ostreatus*	LAPU 53 = ATCC 44309
100	*Pleurotus sajor*-*caju*	FAT
102	*Pleurotus eryngii*	FAT
107	*Pleurotu ostreatus*	FAT = FMC 243
112	*Pleurotus ostreatus*	FCL
127	*Pleurotus pulmonarius*	BIUR T 074
140	*Pleurotus florida*	BIUR T 147
175	*Pleurotus ostreatus*	FCTUA 19
176	*Pleurotus ostreatus*	FCTUA 20–74
177	*Pleurotus ostreatus*	FCTUA 20
178	*Pleurotus sajor*-*caju*	FCTUA 21
234	*Pleurotus ostreatus*	FCTUA 101
237	*Pleurotus sajor*-*caju*	FCTUA 104
238	*Pleurotus eryngii*	FCTUA 105
240	*Pleurotus cystidiosus*	FCTUA 107
246	*Pleurotus ostreatus*	FAT Hokuto YZ
247	*Pleurotus ostreatus*	FAT Mori 39
248	*Pleurotus ostreatus*	FAT Mori 38
I	*Pleurotus* sp.	Environmental isolate^c^
II	*Pleurotus ostreatus*	Commercial mushroom^d^
III	*Pleurotus ostreatus*	Commercial mushroom^e^

^a^FCL, Fungal Collection of Lublin, Department of Biochemistry, Maria Curie-Sklodowska University, Lublin, Poland

^b^LAPU, Laboratory for Anatomy and Physiology of Plants, I.E. Purkyne University, Brno, Slovakia; ATCC, American Type Culture Collection, Rockville, MD; FAT, Professor T. Fukuzumi, Faculty of Agriculture, Agriculture University, Tokyo, Japan; FMC, Section of Mushroom, Forestry and Forest Products Research Institute, Japan; BIUR, Botany Institute II, Regensburg University, Regensburg, Germany; FCTUA, Forest Products Chemistry Laboratory, Agriculture University, Tokyo, Japan

^c^Environmental isolate originating from Lublin, Poland

^d^Commercial oyster derived from the manufacturer of edible mushrooms, L. W. Skrzypczyk, Poland

^e^Commercial oyster derived from the manufacturer of edible mushrooms, H. Kaczmarek, Poland

### Isolation of Total DNA

The fruit body or the mycelia from 40 ml liquid cultures were used for DNA extraction according to the method of Borges et al. [4]. To extract DNA, 0.5–2.0 g of fresh mycelium was ground in liquid nitrogen. The mycelial powder was transferred to a sterile test tube containing 15 ml of cold spermidine–SDS buffer (4 mM spermidine, 10 mM EDTA, 0.1 M NaCl, 0.5 % SDS, 10 mM β-mercaptoethanol, 40 mM Tris–HCl, pH 8.0) and thoroughly shaken for 20 min. The mixture was immediately extracted two times with one volume of double-distilled phenol. Subsequently, the aqueous phase was extracted with one volume of chloroform–isoamyl alcohol (24:1) and centrifuged (10,000×*g*, 10 min, 4 °C). 3 M sodium acetate (pH 5.5) was added to the aqueous phase at the proportion 9:1, respectively. DNA was then precipitated by addition of two volumes of ice-cold 96 % ethanol, and recovered by centrifugation at 10,000×*g* at 4 °C for 10 min. DNA was dried in a vacuum exsiccator (Sigma, USA), redissolved in 1 ml of sterile TE buffer, and stored at −20 °C.

### Ribonuclease Treatment

Extracted nucleic acids were digested with the RNase A (Sigma, USA) according to the manufacturer’s protocol. The final RNase concentration was 10 μg/ml. The reaction mixture was incubated for 30 min at room temperature. The quality and quantity of genomic DNA were accurately measured with spectrophotometric absorbency at 260 and 280 nm, respectively.

### AFLP Analysis

The AFLP reactions were performed as described by Vos et al. [36] with some modifications. Adapters and primers were synthesized by Genset Oligos, France and IBB PAN, Poland.

#### Restriction Ligation

The genomic DNA (1 μg) was digested in the final volume of 30 μl with 20 U of *Pst*I restriction enzyme (Fermentas, Lithuania) for 18 h at 37 °C. The quality and quantity of the digested product were examined by gel electrophoresis, stained with ethidium bromide, and visualized under UV fluorescence as a smear across bromophenol blue.

The double-stranded *Pst*I oligonucleotides adapters were formed in a total volume of 10 μl by incubating 10 μM *Pst*IAF and 10 μM *Pst*IAR adapters at 95 °C for 10 min, following 30 min at room temperature.

The ligation solution containing the double-stranded adapters (10 μl), DNA digested with *Pst*I (850 ng), 5 U T4 DNA polymerase (Fermentas, Lithuania), and 1× T4 ligase buffer (40 mM Tris–HCl, 10 mM MgCl_2_, 10 mM DTT, 0.5 mM ATP, pH 7.8) was incubated for 4 h at 37 °C (25 μl final volume). Ligated DNA was then precipitated with a mixture of 3 M sodium acetate, pH 5.5 and ice-cold 96 % ethanol (1:25) at −18 °C for 30 min to remove unbound adapters. DNA was harvested by centrifugation (14,000 rpm, 4 °C, 20 min) and dried in a vacuum centrifuge. The debris of DNA was dissolved in 50 μl of sterile water and used as a template in the amplification reaction.

#### Nonselective PCR Amplification

Nonselective PCR was performed to check digestion and ligation reactions. PCR was carried out in 20 μl volume containing 5 μl of ligated with double-stranded adapters and purified DNA, 0.2 mM of each dNTP, 1.5 mM MgCl_2_, 0.4 U *Taq* DNA polymerase LC (recombinant) 1 U/μl (Fermentas, Lithuania), 1× PCR buffer (75 mM Tris–HCl, pH 8.8, 20 mM (NH_4_)_2_SO_4_, 0.01 % Tween 20), 750 nM *Pst*IAF primer. Amplifications were carried out in a T-personal thermal cycler (Biometra, Germany) with the conditions as follows: 95 °C for 2 min 30 s followed by 45 cycles of 45 s at 94 °C, 45 s at 54 °C, and 45 s at 72 °C. The final cycle was followed by an additional 10 min at 72 °C.

#### Selective PCR Amplification

PCRs were performed in a 50 μl total volume which consisted of 1× PCR buffer (Fermentas, Lithuania), 2.5 mM MgCl_2_, 0.2 mM of each dNTP, 1 U of *Taq* DNA polymerase LC (recombinant) 1 U/μl (Fermentas, Lithuania), 10 pmol of each primer, 0.5 μl of targeted digested and ligated genomic DNA. All amplification reactions were performed in a T-personal thermal cycler (Biometra, Germany) with the conditions as follows: 94 °C for 2 min 30 s followed by seven cycles of amplification, with annealing temperature decreasing 1 °C/cycle: 94 °C for 30 s, first annealing for 30 s at 60–54 °C or 67–61 °C (annealing temperature depends on primers *T*_m_), 72 °C for 30 s, and next 33 amplification cycles of 94 °C for 45 s, 53 °C or 60 °C (annealing temperature depends on primers *T*_m_) for 45 s, and 72 °C for 45 s. The final cycle was followed by an additional 7 min at 72 °C. The PCR products were stored at 4 °C until further analysis. The adapters and primers employed for AFLP are shown in Table 2.

**Table 2:** List of oligonucleotide primers and adapters

	Adaptor name	Adaptor sequence 5′–3′	Melting temperature (°C)
1	*Pst*IAF	CTCGTAGACTGCGTACATGCA	51
2	*Pst*IAR	TGTACGCAGTCTAC	42

	Primer name	Primer sequence 5′–3′	Melting temperature (°C)
1	*Pst*AAG	GACTGCGTACATGCAGAAG	51.1
2	*Pst*CAA	GACTGCGTACATGCAGCAA	51.1
3	*Pst*G	GACTGCGTACATGCAGG	49.5
4	*Pst*GC	GACTGCGTACATGCAGGC	52.6

### Electrophoresis and Imaging

A 25 μl aliquot of the PCR mixture was combined with 5 μl of loading buffer and the amplicons were separated by electrophoresis in 1.5 % agarose gel in 1× TBE buffer (89 mM Tris base, 89 mM boric acid, 2 mM EDTA, pH 8.0). The electrophoresis was run at 150 V in TBE buffer on a horizontal gel electrophoresis system (Agagel Mini, Biometra) for about 3 h. The gels were stained with ethidium bromide and photographed on a UV transilluminator (Vilber Lourmat, France).

### Data Analysis

Electrophoretograms were analyzed using BIO 1D software (Vilber Lourmat, France). AFLP markers were manually scored as binary data for the presence or absence of fragments between 75 and 3,000 bp. This binary information was used to calculate Jaccard’s pairwise similarity coefficients as implemented in the program FreeTree version 0.9.1.50 [10]. The unweighted pair-group method with arithmetic averages (UPGMA) dendrograms were generated from DNA band patterns using Nei and Li [25] correlation coefficient. The phylogenetic tree was visualized and edited using NTSYSpc software version 2.01. (Exeter Software Co., New York).

## Results

The rare cutting restriction endonuclease *Pst*I and four primers were used in the AFLP analysis to fingerprint the genomes of 18 fungal strains belonging to six species of the genus *Pleurotus*: *P. ostreatus*, *P. sajor*-*caju*, *P. eryngii*, *P. pulmonarius*, *P. florida*, *P. cystidiosus*; two commercially available edible strains of *P. ostreatus* (II and III); and one environmental isolate determined as *Pleurotus* sp. I (Table 1).

The smears obtained in the nonselective PCR amplification proved efficient degradation of DNA by *Pst*I endonuclease. In the selective amplification reactions, all four primers successfully amplified AFLP bands in all the 21 fungi studied. Each of the four primers generated a fingerprint pattern markedly distinct from those of the other primers, even when the primers differed by only one selective nucleotide in the extension. A total of one to three selective bases were found to provide a sufficient complex pattern for the DNA polymorphism analysis (see Table 3). Although a variable number of amplified bands was obtained in the PCR with each primer, all of them generated polymorphic and unambiguously scored fragments, and numbers of scorable fragments.

**Table 3:** AFLP primers used in the analysis of *Pleurotus* sp. strains, number, and range of length of all amplified DNA fragments, number of polymorphic fragments, and percentage of polymorphism

Primer name	Primer’s selective bases	No. of all fragments	Fragment range length	No. of polymorphic fragments	Percentage of polymorphism
*Pst*AAG	AAG	33	95–908	33	100
*Pst*CAA	CAA	18	143–900	18	100
*Pst*G	G	128	75–881	107	83.5
*Pst*GC	GC	192	250–1,750	150	78.1
Sum		371		308	
Average		92.75		77	90.4

The AFLP method applied has provided characteristic genomic markers to differentiate among the analyzed *Pleurotus* strains. The number of the scorable amplicons produced high variation and ranged from 1 to 12. A total of 371 AFLP markers were perceived using four primers. The large number of bands obtained in the PCR with *Pst*G and *Pst*GC primers demonstrate that the AFLP analysis is a robust and efficient method for detecting genomic differences between the strains in the genus *Pleurotus*. Primer *Pst*GC amplified the highest number of fragments (192), while the fewest bands (18) were observed with the primer *Pst*CAA. On average, 92.75 AFLP markers in the size range from 75 to 1,750 bp were amplified per primer. A total of 308 reliable polymorphic bands (90.4 % polymorphism) were observed across all 21 isolates from PCR with four primers, which corresponds to an average of 77 polymorphic bands per primer combination. The main AFLP characteristics for four primers are presented in Table 3.

Pairwise similarities between *Pleurotus* strains (Table 4) were calculated based on the number of polymorphic bands using Jaccard’s similarity coefficient [15]. The genomic similarity between *Pleurotus* species varied from 0.0 (*Pleurotus* sp. I vs. *P. sajor*-*caju* 237 and *P. eryngii* 238) to 0.750 (*P. ostreatus* 246 vs. *P. ostreatus* 248) and on average was 0.378. The average value of Jaccard’s pairwise genomic similarity within the group of *P. ostreatus* was 0.453. Lower variations were found in the groups of *P. sajor*-*caju* and *P. eryngii*: 0.320 and 0.313, respectively.

**Table 4:** Jaccard’s pairwise similarities between analyzed *Pleurotus* sp. strains calculated on the basis of 308 polymorphic bands

	Fungal strain	1	2	3	4	5	6	7	8	9	10	11	12	13	14	15	16	17	18	19	20	21
1	*Pleurotus* sp. I	1.000																				
2	*P. ostreatus* II	0.083	1.000																			
3	*P. ostreatus* III	0.118	0.333	1.000																		
4	*P. ostreatus* 13	0.063	0.368	0.455	1.000																	
5	*P. sajor*-*caju* 100	0.048	0.409	0.423	0.591	1.000																
6	*P. eryngii* 102	0.077	0.438	0.381	0.421	0.333	1.000															
7	*P. ostreatus* 107	0.063	0.444	0.524	0.429	0.458	0.421	1.000														
8	*P. ostreatus* 112	0.118	0.217	0.478	0.391	0.321	0.381	0.455	1.000													
9	*P. pulmonarius* 127	0.095	0.333	0.520	0.440	0.414	0.269	0.440	0.462	1.000												
10	*P. florida* 140	0.050	0.250	0.500	0.417	0.393	0.292	0.417	0.440	0.379	1.000											
11	*P. ostreatus* 175	0.100	0.409	0.542	0.522	0.538	0.333	0.522	0.480	0.577	0.500	1.000										
12	*P. ostreatus* 176	0.045	0.391	0.407	0.636	0.464	0.375	0.440	0.462	0.556	0.481	0.640	1.000									
13	*P. ostreatus* 177	0.063	0.368	0.391	0.304	0.458	0.227	0.500	0.231	0.385	0.308	0.458	0.333	1.000								
14	*P. sajor*-*caju* 178	0.045	0.280	0.357	0.565	0.464	0.269	0.286	0.310	0.400	0.481	0.577	0.680	0.241	1.000							
15	*P. ostreatus* 234	0.083	0.250	0.464	0.393	0.333	0.286	0.393	0.367	0.364	0.387	0.517	0.452	0.345	0.452	1.000						
16	*P. sajor*-*caju* 237	0.000	0.333	0.200	0.217	0.320	0.316	0.400	0.200	0.214	0.280	0.375	0.417	0.333	0.417	0.321	1.000					
17	*P. eryngii* 238	0.000	0.429	0.238	0.333	0.261	0.313	0.333	0.182	0.250	0.217	0.450	0.364	0.333	0.304	0.375	0.467	1.000				
18	*P. cystidiosus* 240	0.125	0.286	0.435	0.409	0.385	0.333	0.348	0.320	0.370	0.346	0.636	0.370	0.348	0.423	0.600	0.261	0.471	1.000			
19	*P. ostreatus* 246	0.143	0.316	0.476	0.526	0.417	0.444	0.526	0.409	0.346	0.375	0.545	0.522	0.450	0.346	0.583	0.350	0.438	0.500	1.000		
20	*P. ostreatus* 247	0.125	0.350	0.375	0.476	0.440	0.333	0.348	0.320	0.370	0.458	0.565	0.480	0.348	0.370	0.481	0.261	0.389	0.524	0.579	1.000	
21	*P. ostreatus* 248	0.143	0.389	0.476	0.611	0.478	0.444	0.526	0.476	0.458	0.435	0.619	0.591	0.318	0.400	0.520	0.286	0.438	0.500	0.750	0.667	1.000

**Fig. 1:**
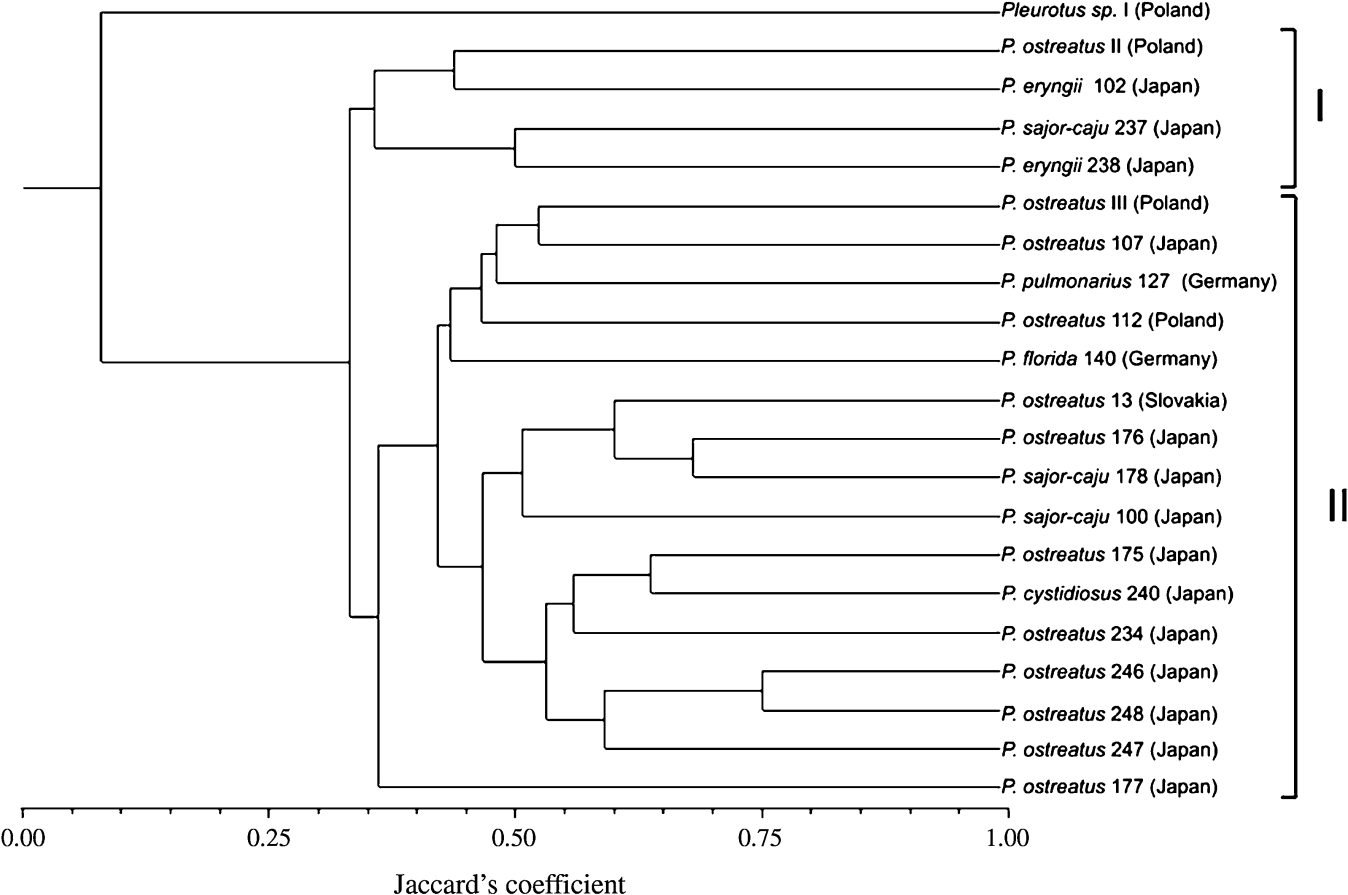
UPGMA tree based on polymorphic AFLP markers showing genomic diversity of fungal strains examined in this study. All the 21 *Pleurotus* sp. strains were grouped into two main clusters (I and II) and one separate lineage. UPGMA cluster analysis was based on Nei and Li’s [25] genetic distance

The genomic relationship between the studied *Pleurotus* strains is presented on the dendrogram constructed with an UPGMA cluster analysis (Fig. 1). Based on the combined data from the AFLP patterns obtained in the PCR with four primers, all the 21 *Pleurotus* species strains were classified into two main clusters at DNA profile similarity of 33 % and one separate lineage occupied by naturally growing *Pleurotus* sp. strain I. Cluster I combined 4 strains, i.e., 2 strains of *P. eryngi*, 1 strain of *P. ostreatus,* and 1 strain of *P. sajor*-*caju*. Cluster II grouped 17 strains classified as *P. ostreatus* (12 strains), *P. sajor*-*caju* (2 strains), *P. cystidiosus* (1 strain), *P. florida* (1 strain), and *P. pulmonarius* (1 strain). *Pleurotus* sp. strain I located on the outskirt of the tree and the other *Pleurotus* sp. strains included in the analysis displayed the AFLP profile similarity level in the range from 0.0 to 14.3 %.

## Discussion

AFLP is capable of simultaneous screening of many different DNA regions distributed randomly throughout the genome after prior DNA digestion with the restriction enzyme [24].

AFLP can be applied to DNAs of many sources and complexity, and it has been reported to be suitable for identification and differentiation of microorganisms at the intraspecies level as well as for determining their genomic relationships [5, 12, 20]. To our knowledge, the AFLP technique has also been used for identification of fungi and analysis of their genomic diversity. Mueller et al. [23] used a simplified AFLP method with only *Pst*I restriction enzyme to detect genomic differences among 14 symbiotic fungi of the fungus-growing ant, *Cyphomyrmex minutus*. Terashima and Matsumoto [30] demonstrated that AFLP analysis is suitable to strain typing with heat-dried fruiting bodies of shitake mushrooms. Urbanelli et al. [33] showed that AFLP distinguish ambiguously three studies by the genus *Pleurotus* ranks: *P. ferulae*, *P. eryngii*, and *P. eryngii* var*. nebrodensis*.

In this study, we used the AFLP method to assess genetic differences among 21 strains representing: *P. ostreatus* (12 strains), *P. sajor*-*caju* (3 strains), *P. eryngi* (2 strains), *P. pulmunarius* (1 strain), *P. florida* (1 strain), *P. cystidiosus* (1 strain), and 1 *Pleurotus* sp. isolated by us from natural environmental samples and classified into the genus *Pleurotus* by morphological features.

By using a combination of four AFLP primers, it was possible to distinguish clearly all the 21 strains of the genus *Pleurotus* at the species and intraspecies levels (Fig. 1). They exhibited very different banding patterns and their pairwise genomic similarities ranged from 0 between *Pleurotus* sp. I—a wild growing fungus and *P. sajor*-*caju* 237 as well as *P. eryngii* 238 (both of them are from culture collections) to 75 % between *P. ostreatus* 176 and *P. sajor*-*caju* 178. The significant genomic diversity of the studied *Pleurotus* sp. strains was demonstrated by cluster analysis of their AFLP profiles (Fig. 1). Each *Pleurotus* strain formed independent lineage on the AFLP tree and they were clustered into four groups and two separate branches at the AFLP genomic profile similarity of 33 %. *Pleurotus* sp. I from the natural environment was located on the outskirt of the AFLP tree (besides *Pleurotus* species from culture collection and two commercial *P. ostreatus* strains). This fact supports the thesis that wild populations of edible mushrooms (well adapted to changing environmental conditions) may be an important source of genomic variability for cultivated mushroom lineages that undergo loss of genomic diversity resulting from man’s selection [33].

Cluster analysis of *Pleurotus* sp. AFLP profiles also revealed that the geographically close *P. ostreatus* strain from Slovakian and Polish culture collections are genomically distant (their AFLP profile similarity is only 39 %). They fall into two different clusters similar to two commercial strains *P. ostreatus* II and III from Polish manufacturers (33 % AFLP profile similarity), which means that they are completely different lineages (Fig. 1).

It is known that in the AFLP analysis, the number of amplicons and the percentage of polymorphic DNA bands are determined by the number of selective nucleotides at the 3′ end of the restriction enzyme primers [16, 36]. This was supported in our studies with DNA of strains of *Pleurotus* genus. Primers with three selective nucleotides amplified fewer restriction fragments compared to those with one and two selective bases (Table 3).

Our results indicated that the AFLP method is very useful for fast, large scale, and reliable typing of strains of *Pleurotus* genus and practical identification of mushroom cultivars.
